# Convergence of HIV and non-communicable disease epidemics: geospatial mapping of the unmet health needs in an HIV hyperendemic community in South Africa

**DOI:** 10.1136/bmjgh-2023-012730

**Published:** 2024-01-04

**Authors:** Diego F Cuadros, Chayanika Devi, Urisha Singh, Stephen Olivier, Alison C Castle, Yumna Moosa, Johnathan A Edwards, Hae-Young Kim, Mark J Siedner, Emily B Wong, Frank Tanser

**Affiliations:** 1 Digital Epidemiology Laboratory, University of Cincinnati, Cincinnati, OH, USA; 2 Africa Health Research Institute, Durban, South Africa; 3 Nelson R Mandela School of Medicine, University of KwaZulu-Natal, Durban, South Africa; 4 Division of Infectious Diseases, Massachusetts General Hospital, Boston, MA, USA; 5 Harvard Medical Shool, Boston, MA, USA; 6 Department of Biostatistics and Bioinformatics, Emory University, Atlanta, GA, USA; 7 International Institute for Rural Health, University of Lincoln, Lincolnshire, UK; 8 Department of Population Health, New York University Grossman School of Medicine, New York, NY, USA; 9 School of Clinical Medicine, College of Health Sciences, University of KwaZulu-Natal, Durban, South Africa; 10 Division of Infectious Diseases, University of Alabama Birmingham, Birmingham, AL, USA; 11 Centre for Epidemic Response and Innovation (CERI), School of Data Science and Computational Thinking, Stellenbosch University, Stellenbosch, South Africa; 12 School of Nursing and Public Health, College of Health Sciences, University of KwaZulu-Natal, Durban, South Africa; 13 Centre for the AIDS Programme of Research in South Africa (CAPRISA), University of KwaZulu-Natal, Durban, South Africa

**Keywords:** HIV, Diabetes, Hypertension, Geographic information systems, Epidemiology

## Abstract

**Introduction:**

As people living with HIV (PLHIV) are experiencing longer survival, the co-occurrence of HIV and non-communicable diseases has become a public health priority. In response to this emerging challenge, we aimed to characterise the spatial structure of convergence of chronic health conditions in an HIV hyperendemic community in KwaZulu-Natal, South Africa.

**Methods:**

In this cross-sectional study, we used data from a comprehensive population-based disease survey conducted in KwaZulu-Natal, South Africa, which collected data on HIV, diabetes and hypertension. We implemented a novel health needs scale to categorise participants as: diagnosed and well-controlled (Needs Score 1), diagnosed and suboptimally controlled (Score 2), diagnosed but not engaged in care (Score 3) or undiagnosed and uncontrolled (Score 4). Scores 2–4 were indicative of unmet health needs. We explored the geospatial structure of unmet health needs using different spatial clustering methods.

**Results:**

The analytical sample comprised 18 041 individuals. We observed a similar spatial structure for HIV among those with combined needs Score 2–3 (diagnosed but uncontrolled) and Score 4 (undiagnosed and uncontrolled), with most PLHIV with unmet needs clustered in the southern urban and peri-urban areas. Conversely, a high prevalence of need Scores 2 and 3 for diabetes and hypertension was mostly distributed in the more rural central and northern part of the surveillance area. A high prevalence of need Score 4 for diabetes and hypertension was mostly distributed in the rural southern part of the surveillance area. Multivariate clustering analysis revealed a significant overlap of all three diseases in individuals with undiagnosed and uncontrolled diseases (unmet needs Score 4) in the southern part of the catchment area.

**Conclusions:**

In an HIV hyperendemic community in South Africa, areas with the highest needs for PLHIV with undiagnosed and uncontrolled disease are also areas with the highest burden of unmet needs for other chronic health conditions, such as diabetes and hypertension. Our study has revealed remarkable differences in the distribution of health needs across the rural to urban continuum even within this relatively small study site. The identification and prioritisation of geographically clustered vulnerable communities with unmet health needs for both HIV and non-communicable diseases provide a basis for policy and implementation strategies to target communities with the highest health needs.

**WHAT IS ALREADY KNOWN ON THIS TOPIC**

- Studies primarily examine individual-level clustering of diseases and use generalised epidemiological measures like prevalence to analyse disease interactions and overlaps.
- Limited understanding of synergistic interactions and patterns between comorbidities at the community level.
- Lack of effective solutions for integrating and analysing data on various diseases in specific populations.

**WHAT THIS STUDY ADDS**

- Here we presented novel insights into the spatial relationship between unmet health needs in chronic infectious and non-communicable diseases in an HIV hyperendemic community in South Africa.
- We found that the high prevalence of HIV coincides with high burden of unmet needs for other diseases, such as diabetes and hypertension, indicating overlapping health challenges.
- Our study emphasises identifying and prioritising regions for effective interventions to improve overall health outcomes in HIV hyperendemic communities.

**HOW THIS STUDY MIGHT AFFECT RESEARCH, PRACTICE OR POLICY**

- Clinical research often focuses on isolated disease-causing agents, neglecting the complex social and ecological contexts of disease emergence and spread.
- Restructuring healthcare delivery to use existing HIV service infrastructure can address other chronic health conditions in HIV hyperendemic communities, improving overall health outcomes.
- Our study supports the need for a holistic approach to addressing disease interactions, particularly in resource-limited settings with high disease burdens.

## Introduction

While the transition of disease burden has predominantly included shifts from infectious diseases to non-communicable diseases (NCDs) globally, numerous studies in South Africa (SA) have also reported the potential emergence of multimorbidity interactions with the convergence of infectious diseases and NCDs.^1–6^ Likewise, life expectancy in people living with HIV (PLHIV) has also rebounded following the increasing access to antiretroviral therapy (ART), resulting in an increased likelihood of comorbidities in this vulnerable population.^7 8^ As a result, detailed epidemiologic studies of colliding HIV and NCDs in SA remain an important and urgent priority to improve the delivery of targeted healthcare.^9^

Recent applications of mapping and spatial analysis have proven to be an effective methodology to uncover health determinants and vulnerable populations at higher risk of chronic health conditions.^10 11^ However, several studies that aimed to uncover the geospatial convergence of different diseases have found no evidence of geographical overlap between the prevalence of HIV and NCDs like cardiovascular diseases and diabetes in SA, at the country and the locale level.^6 12 13^ These results were consistent with other studies that also found no epidemiological overlap between PLHIV and the presence of any NCDs in SA and in other countries in sub-Saharan Africa.^14–16^ Differential local biological, demographic and socioeconomic determinants driving HIV and NCDs microepidemics could be preventing the detection of the overlap among these health chronic conditions in the same communities. Likewise, distinct epidemic stages might generate a mismatch in the epidemic intensities of the diseases that are colliding in the same communities, resulting in lack of awareness about multimorbidity.

Understanding disease progression and the unmet health needs of individuals with chronic conditions is an important step to addressing disease co-occurrence and designing health system response to these. The extent to which health needs of different diseases overlap within individuals and communities would help to uncover disease interactions emerging from multimorbidity associations. In the Vukuzazi study, we assessed the health needs for HIV, hypertension and diabetes in an HIV hyperendemic community in Kwazulu-Natal, SA. In this study, we introduced a novel health needs framework to conceptualise the unmet health needs of communities impacted by the overlapping infectious and noninfectious diseases in the region. Applying this framework, this study found that half of the PLHIV, hypertension or diabetes in this community have unmet health needs, with the greatest concentration of unmet health needs among persons living with NCDs. Furthermore, preliminary spatial visualisations demonstrated unanticipated geographical overlap of unmet health needs that could be used to inform strategies of health service delivery.

In the present study, we build on prior research by conducting in-depth geospatial analyses of the spatial distribution of unmet health needs for HIV, hypertension and diabetes in KwaZulu-Natal, SA. Leveraging a health needs framework previously developed and employing various spatial epidemiology and disease mapping techniques, we examined the geographic convergence of unmet health needs and identified areas where individuals with the greatest needs for HIV and NCDs are concentrated. The identification of these spatial patterns holds important implications for designing prevention and monitoring strategies for individuals with the highest health needs, which could optimise interventions aimed at enhancing services for these vulnerable communities at higher risk of overlapping health conditions.

## Methods

### Data sources

In this cross-sectional study, we analysed data collected in the Vukuzazi Study that was conducted in the demographic surveillance platform of the Africa Centre Demographic Information System (ACDIS)^17^ located in the uMkhanyakude district of KwaZulu-Natal, SA between 2018 and 2020. Sample population and data collection have been described in detail elsewhere.^6 18^ Briefly, eligible adolescent and adult residents, >15 years of age, from the Africa Health Research Institute Demographic Surveillance Area (DSA) in the uMkhanyakude district of KwaZulu-Natal located near the market town of Mtubatuba and covers 438 km^2^, were invited to participate in this cross-sectional survey (online supplemental figure 1).^19^ Participants were visited at their homesteads and invited to participate at a mobile health camp that moved sequentially through the study area during the study period. Anthropomorphic and blood pressure measurements were performed using the WHO STEPS protocol.^20^ Blood was collected for measurement of glycosylated haemoglobin (HbA1c, VARIANT II TURBO Haemoglobin testing system (Bio-Rad, Marnes-la-Coquette, France)) and for HIV (Genscreen Ultra HIV Ag-Ab enzyme immunoassay (Bio-Rad)). For this analysis, we used the health needs definitions described in our previous work.^18^ The health needs scores in this study were calculated based on the health state and associated health needs of the participants. Participants were assigned needs scores ranging from 0 to 4, with Score 0 indicating no health needs and Score 4 indicating unmet health needs. A health needs score for each disease was assigned as follows: (1) diagnosed and optimally treated (Score 1), (2) diagnosed and suboptimally treated (Score 2), (3) diagnosed but not engaged in care (Score 3) and (4) undiagnosed but with a positive screening test in Vukuzazi (Score 4). Hypertension and diabetes were defined based on specific criteria related to diagnosis, treatment and control levels. Detailed description of how hypertension and diabetes were defined along with the health definitions for each score is included in table 1. Briefly, participants who were diagnosed, engaged in care and optimally treated for their condition were assigned a needs Score of 1 and were included in the met needs group. Participants who were diagnosed, engaged in care but suboptimally treated were assigned a needs Score of 2 and were also included in the unmet health needs group. Participants who were diagnosed but not engaged in care were assigned a needs Score of 3 and included in the unmet health needs group. Participants who were undiagnosed but had a positive screening test in the study were assigned a needs Score of 4 and included in the unmet health needs group. For this study, we focused our analyses on the unmet health needs (Scores 2–4), and combined Scores 2 and 3 for individuals diagnosed in need of treatment and Score 4 for individuals with the highest needs.

10.1136/bmjgh-2023-012730.supp1 Supplementary data

**Table 1:** Need score definitions for HIV, diabetes and hypertension

Need score	HIV	Diabetes	Hypertension (HPTN)
Score 1: diagnosed, engaged in care and optimally treated	Known diagnosis of HIV On treatment HIV viral load <40	Known diagnosis of diabetes On treatment HABA1c ≤6.5	Known diagnosis of HPTN On treatment Blood pressure ≤140/≤90 mm Hg
Score 2: diagnosed, engaged in care and suboptimally treated	Known diagnosis of HIV On treatment HIV viral load >40	Known diagnosis of diabetes On treatment HBA1c >6.5	Known diagnosis of HPTN On treatment Blood pressure ≥140/≥90 mm Hg
Score 3: diagnosed, not engaged in care	Known diagnosis of HIV Not on treatment HIV viral load >40	Known diagnosis of diabetes Not on treatment HBA1c >6.5	Known diagnosis of HPTN Not on treatment Blood pressure ≥140/≥90 mm Hg
Score 4: previously undiagnosed, positive screening test	No previous diagnosis of HIV Immunoassay positive HIV viral load >40	No previous diagnosis of diabetes HBA1c ≥6.5	No previous diagnosis of HPTN Blood pressure ≥140/≥90 mm Hg

A general description of the study population and results from the needs scores estimated in the Vukuzazi study can be found elsewhere.^18^ Briefly, the study enrolled more female participants (67.8%) than male participants (32.2%). Overall, the Vukuzazi study found that approximately half of the people living with chronic disease in the community had unmet health needs. Specifically, 54.9% of the participants had at least one of the three health conditions measured. Of these individuals, 61.7% had HIV, 46.6% had hypertension and 17.6% had diabetes. The distribution of health needs varied among individuals with different health conditions. For individuals with a single health condition, 50.1% had their health needs met, while 49.9% had at least one unmet health need. Among those with unmet health needs, 36.5% required treatment optimisation, 25.9% were diagnosed but not engaged in care and 37.6% were undiagnosed and in need of further diagnostic testing, engagement in care, treatment optimisation, provision of chronic medication and routine monitoring. The distribution of health needs also varied by age. Younger participants had a greater need for diagnosis, with 66.7% of participants aged 15–29 years with comorbid HIV and hypertension needing a diagnosis compared with 41.1% of participants aged 30–49 years and 17.1% of participants aged 50 years or older with the same combination of conditions. This study follows the Strengthening the Reporting of Observational Studies in Epidemiology reporting guideline.^21^

### Geospatial analyses

Participants included in the study were geolocated to their respective homesteads of residence using the comprehensive geographic information system. To protect the confidentiality of the participants, a geographical random error was introduced to the geographical coordinates of each homestead included in the study.^22^ The geospatial distribution of the prevalence of needs Scores 2 and 3 combined (individuals diagnosed in need of treatment) and needs Score 4 (individuals with the highest needs) for each of the three diseases included in the study was assessed by the generation of continuous surface maps using a standard Gaussian kernel interpolation method (with a search radius of 3 km), which has been used and validated in this population for mapping multiple HIV outcomes in the area of study,^22^ and the spatial structure of the prevalence of these unmet needs scores for each disease was identified using optimised hotspot analysis, which identifies areas with high (hotspots) or low (coldspots) prevalence of these unmet needs.^23 24^ We identified geospatial associations between a combination of the needs score of two diseases applying spatial bivariate and multivariate analyses using the geospatial GeoDa environment.^25^ First, spatial correlations between a combination of the needs score of two diseases were identified using bivariate local indicators of spatial association (LISA). The bivariate LISA statistics identified significant spatial clustering based on the degree of linear association between the prevalence of the needs score of one disease at a given location and the prevalence of the needs score of the other disease at neighbouring locations.^26^ Maps were generated illustrating the locations with statistically significant associations and the type of spatial association between the needs score of both diseases (ie, high–high, low–low, low–high and high–low). Second, we implemented a method previously used to identify multivariable spatial associations of health determinants using K-means clustering analysis^13 27–30^ to identify the spatial relationship between the distribution of the prevalence of the highest needs (needs Score 4) of all three diseases, HIV, diabetes and hypertension. K-means is a partitioning clustering method in which the data are partitioned into *k* groups (ie, fourth groups). In this clustering method, the *n* observations are grouped into *k* clusters such that the intra-cluster similarity is maximised (or dissimilarity minimised), and the between-cluster similarity minimised (or dissimilarity maximised). A further detailed description of these geospatial methods can be found elsewhere.^31 32^ Percentages for the prevalence of needs score 4 for each disease in every cluster identified were reported. Maps of the results were generated using ArcGIS Pro 3.1.0.^33^

## Results

**Figure 1:**
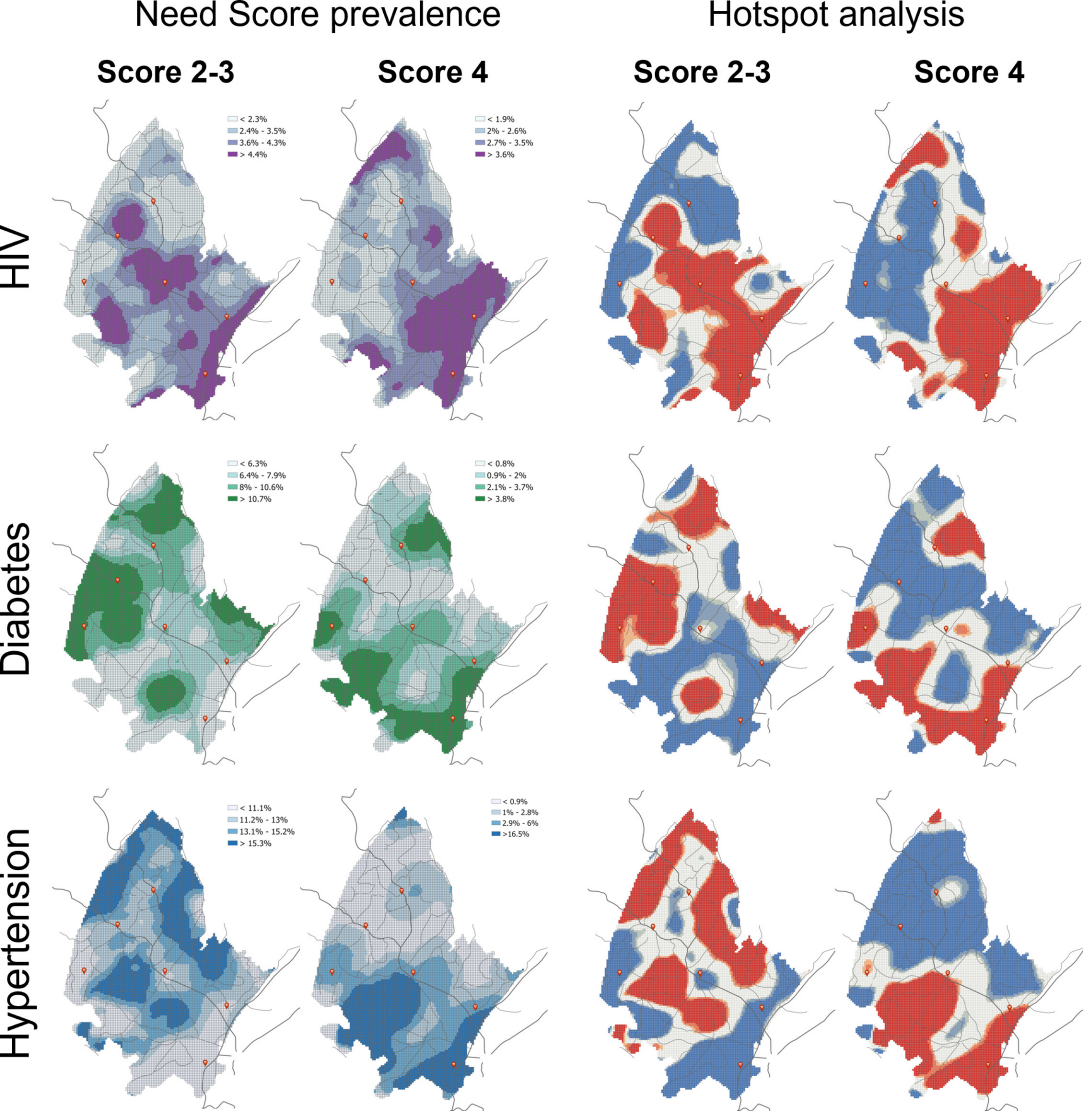
Spatial structure of the prevalence of need Scores 2 and 3 combined and need Score 4 for each disease included in the study: HIV, diabetes and hypertension. Maps on the right illustrate the results of the optimised hotspot analysis for each disease, with areas in red illustrating the identified hotspots, whereas areas in blue correspond to the location of the coldspots. Dark lines illustrate the main roads and red dots illustrate the location of the main healthcare facilities within the surveillance area.

**Figure 2:**
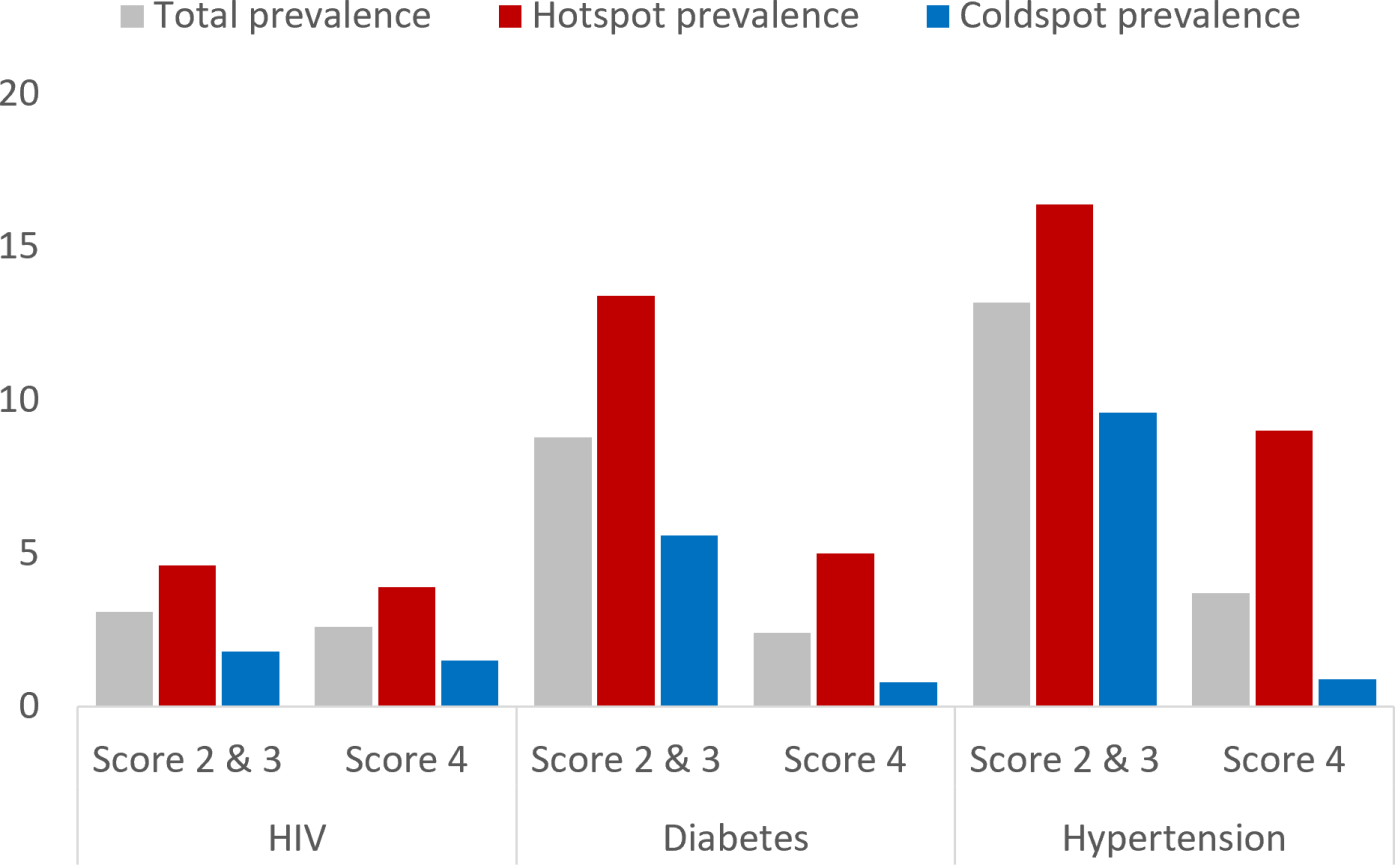
Estimated spatially smoothed prevalence for the need Scores 2 and 3 combine and, need Score 4 for all three diseases included in the study: HIV, diabetes and hypertension. Grey bars illustrate the total prevalence for each disease, whereas red bars and blue bars illustrate the prevalence within the hotspot and the coldspot, respectively.

A total of 18 041 individuals were enrolled in the Vukuzazi study, representing 50% of the 36 097 eligible residents of the DSA. We estimated that 54.9% of the participants had at least one of the three diseases included in the study. Of those individuals with at least one disease, 61.7% had HIV, 17.6% had diabetes and 46.6% had hypertension. Maps in figure 1 illustrate the spatial distribution of need Scores 2 and 3 combined and need Score 4 for each disease. Need Scores 2 and 3 and need Score 4 had similar spatial distribution for HIV, with a higher concentration of individuals with these high needs concentrated in the southern part of the surveillance area. Conversely, a high prevalence of need Scores 2 and 3 for diabetes and hypertension was mostly distributed in the centre and northern part of the surveillance area. Furthermore, a high prevalence of need Score 4 for diabetes and hypertension was mostly distributed in the southern part of the surveillance area. Estimated spatially smoothed prevalence for the different health need scores for all three diseases within the hotspots and coldspots identified is compared in figure 2. Prevalence of need Score 4 for HIV within the hotspot was 3.9% compared with 1.5% within the identified coldspots. For diabetes, prevalence of need Score 4 within the hotspot was 5.0% compared with 0.8% within the coldspots, whereas prevalence of Score 4 for hypertension within the hotspot was 9.0% compared with 0.9% within the identified coldspots.

**Figure 3:**
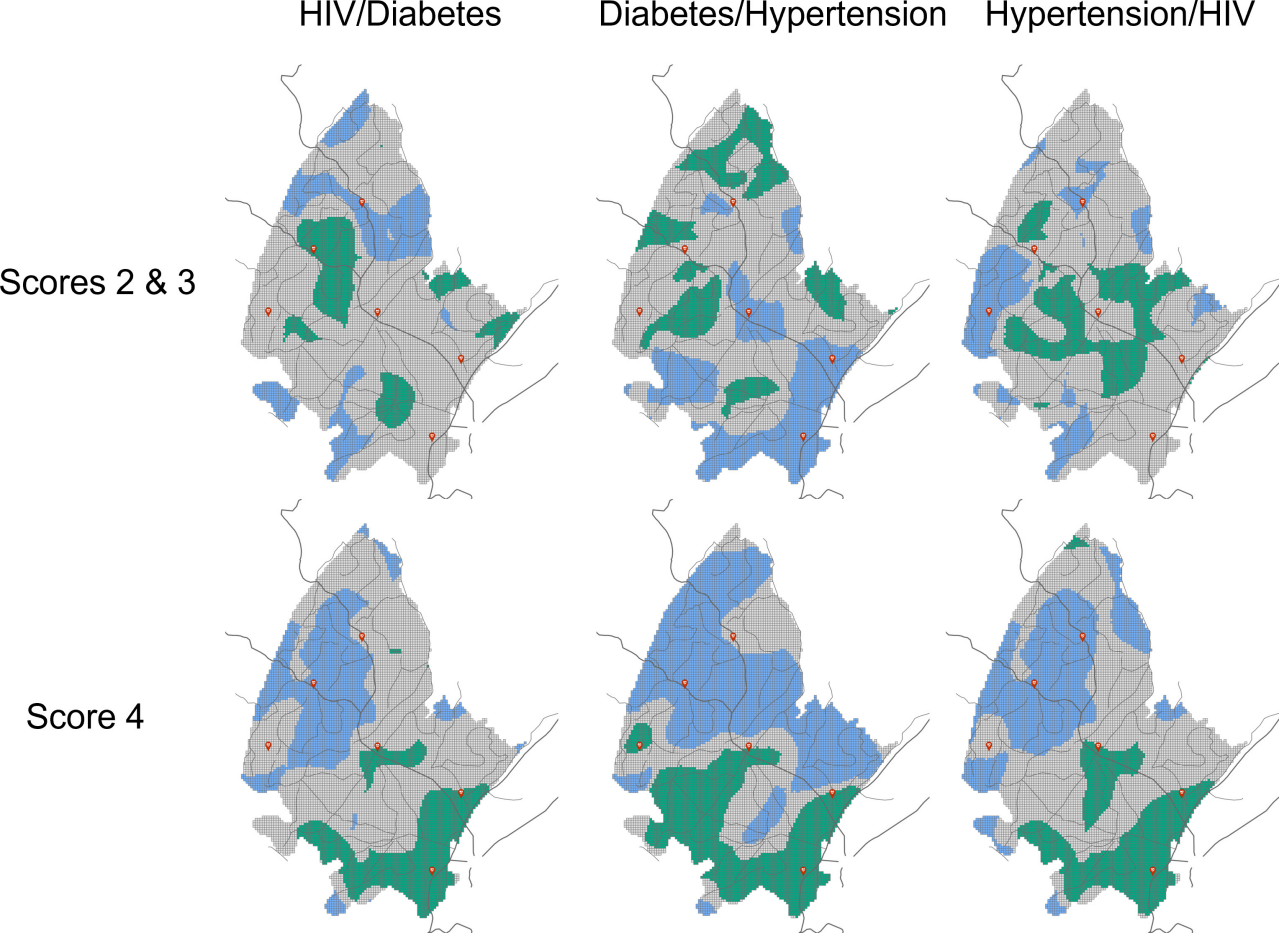
Linear association of spatial autocorrelation (LISA) analysis for the combination between the need scores for each disease included in the study. Green colour indicates high–high association, whereas blue colour indicates areas with low–low associations. Grey colour indicated non-significant association. Dark lines illustrate the main roads and red dots illustrate the location of the main healthcare facilities within the surveillance area.

**Figure 4:**
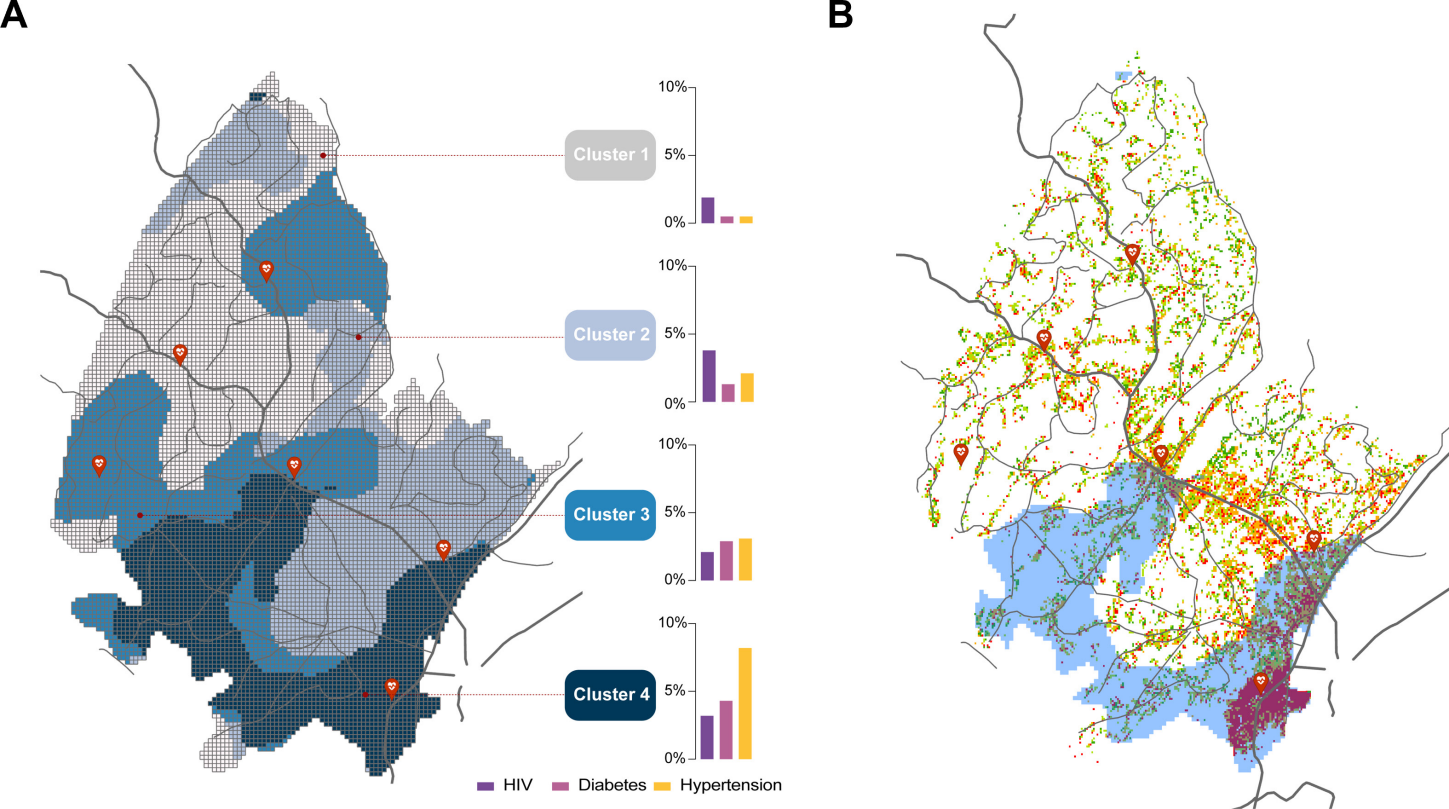
Multivariate K-means clustering analysis for need Score 4 of all three diseases combined (A). Cluster 4 with the highest prevalence of need Score 4 for diabetes and hypertension, and the second highest prevalence of need Score 4 for HIV is illustrated in dark blue. Population density distribution within the surveillance area and within Cluster 4 (B). Red areas illustrate highly dense population locations. Dark lines illustrate the main roads and red dots illustrate the location of the main healthcare facilities within the surveillance area.

Bivariate LISA analysis did not identify a clear spatial pattern of associations between the combination need Scores 2 and 3 for all diseases (maps on top in figure 3). Conversely, there was a significant association of overlapping Score 4 for all combination of diseases, which was located in the southern part of the surveillance area (green areas in maps of the bottom of figure 3). Multivariable K-means clustering analysis of need Score 4 was consistent with this result and identified a cluster (Cluster 4, dark blue areas in map in figure 4A) located in the southern part of the surveillance area. This cluster had the highest prevalence of need Score 4 for diabetes (4.3%) and hypertension (8.2%), and the second highest prevalence of need Score 4 for HIV (3.2%). This cluster occupied 23.2% of the surveillance area and 52.1% (52 991 of the 101 627 individuals living in the DSA)^19^ reside within this cluster with high prevalence of need Score 4 identified (figure 4B).

## Discussion

In this study, we aimed to investigate the spatial distribution of health needs related to chronic health conditions, such as HIV, hypertension and diabetes, in a community facing a hyperendemic HIV epidemic in SA. Our results showed that individuals with the highest health needs for these three diseases clustered in the same location within the surveillance area, indicating a significant overlap of unmet health needs. Multivariable clustering analysis confirmed this finding, revealing a cluster of individuals with the highest needs for all three diseases in the southern part of the surveillance area. This area, covering about 23% of the surveillance region, had the highest prevalence of undiagnosed and not engaged hypertension and diabetes care, and the second highest prevalence of PLHIV with the highest health needs. Moreover, this cluster was located in an urban and peri-urban area with high population density, where over 50% of the population in the community resided. These findings suggest the urgent need for an integrated approach that addresses multimorbidity in delivering care to this vulnerable community. Our study highlights an opportunity to leverage the existing HIV service infrastructure developed over the past few decades to address the needs of other chronic health conditions in these communities and to target their specific health needs.^34 35^

The distribution of categories of unmet health needs for PLHIV had similar spatial distributions, such that PLHIV engaged in care but suboptimally treated (Score 2), those not engaged in care (Score 3) and those undiagnosed (Score 4) were spatially overlapping in the urban and peri-urban southern part of the study surveillance area. Distributions of health needs for people with hypertension and diabetes showed unique geospatial distributions. Individuals with suboptimally treated or not engaged in care for hypertension were mainly distributed in the more rural centre and northern parts of the surveillance area, whereas hypertensive individuals with the highest needs (Score 4) were clustered in the southern part of the surveillance area. Similarly, individuals with suboptimally treated or not engaged in care for diabetes were mostly concentrated in the eastern and northern part of the surveillance area, whereas diabetic individuals with undiagnosed disease and not engaged in care showed similar distribution to the undiagnosed HIV and hypertensive patients and clustered in the southern part of the surveillance area.

Preliminary geospatial results from the Vukuzazi study using disease prevalence estimates found no spatial overlaps among the three chronic health conditions assessed in this study, HIV, hypertension and diabetes.^6^ Similarly, results at the national level suggested a lack of geographical overlap among disease prevalence estimations of HIV and other NCDs including cardiovascular diseases and diabetes in SA.^13^ These findings can be the result of disparities in the epidemic stages of these diseases disproportionately affecting different communities with distinct socioeconomic and demographic profiles. However, the implementation of a more granular epidemiological measures, like disease stages and the health needs emerging from these states, can uncover spatial associations and convergences of diseases affecting the same communities. Not all communities are experiencing the same health needs, and the identification of these uneven epidemiological landscapes could support the design and implementation of prevention and control interventions targeting the specific health needs emerging from the affected community.

In this study, we identified different patterns of distribution for the various health needs for HIV, diabetes and hypertension. We observed that consistent spatial patterns for all health needs were only occurring for HIV. PLHIV with suboptimal treatment, not engaged in care or undiagnosed, clustered in the urban and peri-urban southern part of the surveillance area, a location that is also experiencing a high burden of the HIV epidemic with most of the HIV prevalence and incidence being concentrated in this area.^36^ Aligned with the 95-95-95 UNAIDS targets,^37^ these results highlight the need for the intensification of all 95’s targets in the identified area, with an increase in testing to identify those undiagnosed HIV-positive individuals, expansion of ART coverage to include those underserved individuals not engaged in care, and to sustain ART services to improve outcomes in individuals suboptimally treated. However, it is important to note that PLHIV had the highest percentage of the population with the needs met in this HIV hyperendemic community,^18^ with more than 78% of the HIV-positive participants having their health needs met (diagnosed, engaged in care and optimally treated). This result is consistent with the healthcare spending allocations of the country that are prioritising the HIV epidemic and remains directed towards HIV interventions like ART.^38–40^ Conversely, there are limited finances available for other chronic health conditions.^40–42^ Consequently, results from the Vukuzazi study found a much lower percentage of the population with their health needs met for the NCDs included in the study, with only 6.9% of participants with diabetes and 41.8% of participants with hypertension having their health needs fully met. Likewise, we identified clustered areas where about 15% of the total population had diabetes, and 17% had hypertension, with a disease suboptimally treated or are participants not engaged in care, compared with the only 5% for PLHIV.

By assessing the spatial overlap of unmet health needs for each disease, we delve into an exploration of multimorbidity at the community level. This approach shifts the focus from individual patients to entire communities, providing a broader perspective on how diseases coexist and interact within specific regions. Understanding multimorbidity at the community level offers a holistic view of the health landscape, revealing patterns, hotspots and potential socioeconomic or environmental drivers that might be missed when focusing solely on individual cases. This community-centric approach is particularly valuable for designing public health interventions. Instead of tailoring strategies for individual patients, interventions can be crafted to address the collective needs of entire communities, ensuring that resources, awareness campaigns and treatment programmes are directed where they can have the most substantial impact. By targeting areas with pronounced spatial overlaps of unmet health needs, public health initiatives can achieve broader reach, greater efficiency and more sustainable outcomes, making the community level approach a strategic choice for tackling the multifaceted challenges of multimorbidity.

Moreover, results from ecological studies that highlight these overlaps can be instrumental in designing targeted control interventions. Instead of implementing broad, one-size-fits-all strategies, interventions can be tailored to address the unique challenges of regions suffering from multiple disease burdens. For areas facing with both HIV and diabetes or hypertension, integrated healthcare facilities can be established to provide comprehensive care for all conditions. The established infrastructure for HIV care, which has been refined over years of addressing the epidemic, presents a valuable foundation that can be leveraged to manage other diseases, especially chronic conditions like diabetes and hypertension. Given the systems, expertise and outreach mechanisms already in place for HIV care, these can be adapted and expanded to incorporate treatment and awareness strategies for other chronic diseases. By integrating care for diabetes and hypertension within the existing HIV care framework, we can ensure a holistic approach to patient health, streamline resources and capitalise on the trust and familiarity that communities have with HIV care centres. This integrated model not only enhances the efficiency of healthcare delivery but also offers a comprehensive solution to address the growing challenge of multimorbidity.

It is important to note that the proximity and accessibility of health services can influence the diagnosis and management of diseases. If healthcare facilities are distant or hard to reach, individuals might delay or avoid seeking care, leading to undiagnosed or uncontrolled conditions. Likewise, the type and quality of health services available can impact the effectiveness of disease management. For instance, specialised clinics or centres with advanced equipment and trained personnel might offer better care for specific conditions compared with general clinics. Given the multimorbidity patterns observed in this study, there is a need for healthcare services that offer integrated care for multiple conditions. If a person with HIV also has diabetes, they should be able to receive care for both conditions in a coordinated manner. Furthermore, health services play a crucial role in educating the community about disease prevention, early detection and management. The presence of active health campaigns or outreach programmes can influence community awareness and health-seeking behaviours. While our study provides valuable insights into the geospatial distribution of health needs, the direct influence of the location or type of health services was not elaborated on. However, the availability, accessibility and quality of health services can significantly impact the health outcomes of a community, especially in regions with high disease burdens. For that reason, further studies assessing the location, type and quality of health services are warranted.

### Limitations

There are several limitations worth noting. Although a high rate of enrolment for a population-based multi-disease study was reported,^43^ non-systematic non-response in the Vukuzazi study including lower rates of enrolment by men could have biased the estimation of the health needs and their associations in directions hard to anticipate based on known differences between the sampled and unsampled population.^44^ In the Vukuzazi study, elevated blood pressure and blood glucose were based on measurements conducted on a single day. Therefore, we acknowledge that people who screened positive for diabetes and hypertension required confirmatory testing prior to confirmation of diagnosis, and that this testing could rule out disease requiring immediate treatment. Thus, we may have overestimated the burden of undiagnosed disease.^45^ Furthermore, only three chronic disease conditions were considered in this study, and it must be taken into consideration that there are several other additional NCDs which have not been included in the Vukuzazi study including cancer, chronic respiratory diseases, mental health and other NCDs, limiting our ability to draw comprehensive conclusions about population health and multimorbidity. Likewise, the data analysed in this study are cross-sectional and so do not allow causal inference on their impact on morbidity and mortality.

While the ACDIS cohort provides detailed and unique data, generalising its findings to other settings in Africa requires caution. Therefore, there are some considerations for the generalisations of the findings of this study. Different regions in Africa might have varying socioeconomic, cultural and health system contexts. These differences can influence health outcomes and the effectiveness of interventions. Moreover, the prevalence and dynamics of diseases like HIV and TB can vary across regions, and findings from one setting might not directly apply to another. Furthermore, interventions tested and found effective in the ACDIS cohort might require modifications when implemented in other settings, considering local resources, infrastructure, and cultural distinctions.

Despite these limitations, the ACDIS cohort, in which data for the Vukuzazi study was collected, provides comprehensive epidemiological data from rural KwaZulu-Natal, a region significantly affected by the HIV epidemic and other health challenges. This data, while specific to KwaZulu-Natal, offers insights that can be applied to other African regions with similar socioeconomic and health infrastructure characteristics. Our multimorbidity study using Vukuzazi data highlights the spatial distribution of chronic health conditions, notably HIV, diabetes and hypertension. Patterns observed, such as specific areas with high health needs, suggest socioeconomic and cultural influences on health outcomes. Likewise, recognising spatial clusters with significant health needs can direct resource allocation, ensuring regions facing the most severe health challenges receive appropriate medical resources. The overlap of conditions like HIV, diabetes and hypertension underscores the need for integrated healthcare facilities. Using ACDIS data, national health strategies can prioritise clinics offering comprehensive care for these conditions, enhancing patient experience and efficiency. Moreover, our results can shape health awareness campaigns, especially in areas resembling the southern parts of the surveillance region. Emphasising the inter-relation of these chronic diseases and promoting regular screenings becomes crucial. Our findings along with the findings from the other Vukuzazi studies also highlight the need for training healthcare professionals in managing coexisting chronic conditions, leading to specialised training initiatives. These insights can influence national health policies, emphasising integrated services for chronic diseases and comprehensive health insurance coverage. In essence, by analysing data from specific cohorts like ACDIS and understanding the driving factors behind observed health trends, SA can develop evidence-based interventions addressing multimorbidity challenges. The geospatial approach used here offers a model for other regions to map their health landscapes, ensuring interventions are evidence-driven and contextually relevant.

## Conclusions

In this study, we identified distinct geospatial patterns in the distribution of health needs for HIV, diabetes and hypertension in an HIV hyperendemic South African community. The identification of these patterns is essential for enhancing healthcare services tailored to the specific needs of these communities. In particular, there is a need to improve access to adequate care for individuals already diagnosed with these chronic diseases but lacking appropriate care. Notably, we found that individuals with the highest health needs for all three diseases were concentrated in urban and peri-urban communities located in the southern part of the surveillance area, indicating the potential benefits of integrating chronic disease management models developed for HIV care into the management of other chronic health conditions.^39^ Such integration could be a critical step towards improving healthcare delivery for vulnerable communities experiencing overlapping health conditions.

To achieve this goal, resource allocation should be based on geotargeting and tailored to the specific epidemiological needs of the affected community. Our findings underscore the importance of strategic resource allocation and efficient use of resources to improve health outcomes for populations with complex health needs. Therefore, our study provides valuable insights into the potential benefits of developing geographically targeted interventions aimed at improving access to health services and reducing the burden of overlapping health conditions.

10.1136/bmjgh-2023-012730.supp2 Supplementary data

## Data Availability

Data are available in a public, open access repository. All data are available in public repositories: https://data.ahri.org/index.php/home.

## References

[1] Kabudula CW, Houle B, Collinson MA, et al. Progression of the epidemiological transition in a rural South African setting: findings from population surveillance in agincourt, 1993-2013. BMC Public Health 2017;17:424. 10.1186/s12889-017-4312-x 28486934 PMC5424387

[2] Oni T, Youngblood E, Boulle A, et al. Patterns of HIV, TB, and non-communicable disease multi-morbidity in peri-urban South Africa- a cross sectional study. BMC Infect Dis 2015;15:20. 10.1186/s12879-015-0750-1 25595711 PMC4300166

[3] Peer N. The converging burdens of infectious and non-communicable diseases in rural-to-urban migrant sub-Saharan African populations: a focus on HIV/AIDS, tuberculosis and cardio-metabolic diseases. Trop Dis Travel Med Vaccines 2015;1:6. 10.1186/s40794-015-0007-4 28883938 PMC5526364

[4] Seedat Y, Ali A, Ferdinand KC. Hypertension and cardiovascular disease in the sub-Saharan African context. Ann Transl Med 2018;6:297. 10.21037/atm.2018.06.45 30211185 PMC6123213

[5] Ciccacci F, Tolno VT, Doro Altan AM, et al. Noncommunicable diseases burden and risk factors in a cohort of HIV+ elderly patients in Malawi. AIDS Res Hum Retroviruses 2019;35:1106–11. 10.1089/AID.2019.0125 31468993

[6] Wong EB, Olivier S, Gunda R, et al. Convergence of infectious and non-communicable disease epidemics in rural South Africa: a cross-sectional, population-based multimorbidity study. Lancet Glob Health 2021;9:e967–76. 10.1016/S2214-109X(21)00176-5 34143995 PMC8220132

[7] Levitt NS, Steyn K, Dave J, et al. Chronic noncommunicable diseases and HIV-AIDS on a collision course: relevance for health care delivery, particularly in low-resource settings—insights from South Africa. Am J Clin Nutr 2011;94:1690S–1696S. 10.3945/ajcn.111.019075 22089433 PMC3226022

[8] Magodoro IM, Esterhuizen TM, Chivese T. A cross-sectional, facility based study of comorbid non-communicable diseases among adults living with HIV infection in Zimbabwe. BMC Res Notes 2016;9:379. 10.1186/s13104-016-2187-z 27484005 PMC4969634

[9] High KP, Brennan-Ing M, Clifford DB, et al. HIV and aging: state of knowledge and areas of critical need for research. A report to the NIH office of AIDS research by the HIV and aging working group. J Acquir Immune Defic Syndr 2012;60 Suppl 1:S1–18. 10.1097/QAI.0b013e31825a3668 22688010 PMC3413877

[10] Noble D, Smith D, Mathur R, et al. Feasibility study of geospatial mapping of chronic disease risk to inform public health commissioning. BMJ Open 2012;2:e000711. 10.1136/bmjopen-2011-000711 PMC3282296 22337817

[11] Casper M, Kramer MR, Peacock JM, et al. Population health, place, and space: spatial perspectives in chronic disease research and practice. Prev Chronic Dis 2019;16:E123. 10.5888/pcd16.190237 31489834 PMC6745927

[12] Egede LE, Walker RJ, Monroe P, et al. HIV and cardiovascular disease in sub-Saharan Africa: demographic and health survey data for 4 countries. BMC Public Health 2021;21:1122. 10.1186/s12889-021-11218-5 34118912 PMC8196536

[13] Cuadros DF, Moreno CM, Tomita A. Geospatial assessment of the convergence of communicable and non-communicable diseases in South Africa. medRxiv 2001. 10.1101/2023.03.01.23286636 PMC10540575 37781137

[14] Magodoro IM, Okello S, Dungeni M, et al. Association between HIV and prevalent hypertension and diabetes mellitus in South Africa: analysis of a nationally representative cross-sectional survey. Int J Infect Dis 2022;121:217–25. 10.1016/j.ijid.2022.05.035 35597557 PMC9337715

[15] Bailey SL, Ayles H, Beyers N, et al. Diabetes mellitus in Zambia and the Western Cape province of South Africa: prevalence, risk factors, diagnosis and management. Diabetes Res Clin Pract 2016;118:1–11. 10.1016/j.diabres.2016.05.001 27485851 PMC4994576

[16] Chang AY, Gómez-Olivé FX, Payne C, et al. Chronic multimorbidity among older adults in rural South Africa. BMJ Glob Health 2019;4:e001386. 10.1136/bmjgh-2018-001386 PMC6688670 31423345

[17] Herbst K, Harling G, Siedner M, et al. Africa health research institute surveillance dataset repository. 2023. Available: 10.23664/AHRI.SEH2023

[18] Singh U, Olivier S, Cuadros D, et al. The met and unmet health needs for HIV, hypertension, and diabetes in rural Kwazulu-natal, South Africa: analysis of a cross-sectional multimorbidity survey. Lancet Glob Health 2023;11:e1372–82. 10.1016/S2214-109X(23)00239-5 37591585 PMC10447220

[19] Gunda R, Koole O, Gareta D, et al. Cohort profile: the Vukuzazi (‘wake up and know Yourself’In isiZulu) population science programme. Int J Epidemiol 2022;51:e131–42. 10.1093/ije/dyab229 34849923 PMC9189966

[20] World Health Organization. The WHO STEPwise approach to noncommunicable disease risk factor surveillance (STEPS) - instrument. Geneva, Switzerland,

[21] Elm E von, Altman DG, Egger M, et al. The strengthening the reporting of observational studies in epidemiology (STROBE) statement: guidelines for reporting observational studies. BMJ 2007;335:806–8. 10.1136/bmj.39335.541782.AD 17947786 PMC2034723

[22] Tanser F, Bärnighausen T, Cooke GS, et al. Localized spatial clustering of HIV infections in a widely disseminated rural South African epidemic. Int J Epidemiol 2009;38:1008–16. 10.1093/ije/dyp148 19261659 PMC2720393

[23] Getis A, Ord JK. The analysis of spatial association by use of distance statistics. In: Perspectives on spatial data analysis. Springer, 2010: 127–45. 10.1007/978-3-642-01976-0

[24] Ord JK, Getis A. Local spatial autocorrelation statistics: distributional issues and an application. Geogr Anal 1995;27:286–306. 10.1111/j.1538-4632.1995.tb00912.x

[25] Anselin L, Li X, Koschinsky J. From the desktop to an ecosystem for exploring spatial data. Geogr Anal 2022;54:439–66. 10.1111/gean.12311

[26] Anselin L. Local indicators of spatial association—LISA. Geogr Anal 1995;27:93–115. 10.1111/j.1538-4632.1995.tb00338.x

[27] Cuadros DF, Moreno CM, Musuka G, et al. Association between vaccination coverage disparity and the dynamics of the COVID-19 Delta and Omicron waves in the US. Front Med (Lausanne) 2022;9:898101. 10.3389/fmed.2022.898101 35775002 PMC9237603

[28] Requia WJ, Koutrakis P, Roig HL, et al. Association between vehicular emissions and cardiorespiratory disease risk in Brazil and its variation by spatial clustering of socio-economic factors. Environ Res 2016;150:452–60. 10.1016/j.envres.2016.06.027 27393825

[29] Chandu VC. Identification of spatial variations in COVID-19 epidemiological data using K-means clustering algorithm: a global perspective. MedRxiv 2020. 10.1101/2020.06.03.20121194

[30] Asare K, Tomita A, Garrett N, et al. Depression onset and its association with community HIV prevalence: a geospatial and panel analyses of nationally representative South African data, 2015-2017. J Affect Disord Rep 2022;10:100433. 10.1016/j.jadr.2022.100433

[31] Anselin L, Syabri I, Kho Y. Geoda: an introduction to spatial data analysis. Geogr Anal 2006;38:5–22. 10.1111/j.0016-7363.2005.00671.x

[32] Murray AT, Grubesic TH. Exploring spatial patterns of crime using non-hierarchical cluster analysis. In: Crime modeling and mapping using geospatial technologies. 2013: 105–24. 10.1007/978-94-007-4997-9

[33] ESRI. ArcGIS Pro.x. Redlands. CA, USA, 2020.

[34] Nugent R, Barnabas RV, Golovaty I, et al. Costs and cost-effectiveness of HIV/noncommunicable disease integration in Africa: from theory to practice. AIDS 2018;32 Suppl 1:S83–92. 10.1097/QAD.0000000000001884 29952794 PMC6503960

[35] Matanje Mwagomba BL, Ameh S, Bongomin P, et al. Opportunities and challenges for evidence-informed HIV-noncommunicable disease integrated care policies and programs: lessons from Malawi, South Africa, Swaziland and Kenya. AIDS 2018;32 Suppl 1:S21–32. 10.1097/QAD.0000000000001885 29952787

[36] Tanser F, Vandormael A, Cuadros D, et al. Effect of population viral load on prospective HIV incidence in a hyperendemic rural African community. Sci Transl Med 2017;9:eaam8012. 10.1126/scitranslmed.aam8012 29237762 PMC6435884

[37] Ehrenkranz P, Rosen S, Boulle A, et al. The revolving door of HIV care: revising the service delivery cascade to achieve the UNAIDS 95-95-95 goals. PLoS Med 2021;18:e1003651. 10.1371/journal.pmed.1003651 34029346 PMC8186775

[38] Jailobaeva K, Falconer J, Loffreda G, et al. An analysis of policy and funding priorities of global actors regarding noncommunicable disease in low- and middle-income countries. Global Health 2021;17:68. 10.1186/s12992-021-00713-4 34187499 PMC8240078

[39] Manne-Goehler J, Montana L, Gómez-Olivé FX, et al. The ART advantage: Healthcare utilization for diabetes and hypertension in rural South Africa. J Acquir Immune Defic Syndr 2017;75:561–7. 10.1097/QAI.0000000000001445 28696346 PMC5516957

[40] Blecher MS, Kollipara A, Daven J, et al. HIV and AIDS financing in South Africa: sustainability and fiscal space. S Afr Health Rev 2016;2016:203–19.

[41] Yuyun MF, Sliwa K, Kengne AP, et al. Cardiovascular diseases in sub-Saharan Africa compared to high-income countries: an epidemiological perspective. Glob Heart 2020;15:15. 10.5334/gh.403 32489788 PMC7218780

[42] Juma PA, Mohamed SF, Matanje Mwagomba BL, et al. Correction to: non-communicable disease prevention policy process in five African countries. BMC Public Health 2018;18:1112. 10.1186/s12889-018-5993-5 30205829 PMC6131736

[43] Centers for Disease Control. NHANES response rates and population totals, 2017-2018. Atlanta, Georgia: National Center for Health Statistics,

[44] Wong EB, Olivier S, Gunda R, et al. Convergence of infectious and non-communicable disease epidemics in rural South Africa: a population-based Multimorbidity study. Lancet Glob Health 2021;9:e967–76. 10.1016/S2214-109X(21)00176-5 34143995 PMC8220132

[45] Olivier S, Murray T, Matthews P, et al. Pitfalls of single measurement screening for diabetes and hypertension in community-based settings. Glob Heart 2021;16:79. 10.5334/gh.1083 34900570 PMC8641532

