# Supplementary material for: Convergence of HIV and non-communicable disease epidemics: geospatial mapping of the unmet health needs in an HIV hyperendemic community in South Africa
Source: BMJ Glob Health. 2024 Jan 4;9(1):e012730. doi: 10.1136/bmjgh-2023-012730 (PMC10773360; doi:10.1136/bmjgh-2023-012730)

Supplementary Materials for: Convergence of HIV and non-communicable disease epidemics: Geospatial mapping of the unmet health needs in a HIV hyperendemic community in South Africa

Diego F Cuadros[1*], Chayanika Devi[1], Urisha Singh[2,3], Stephen Olivier[2], Alison Castle[2,4,5], Yumna Moosa[2], Johnathan A Edwards[6,7,8], Hae-Young Kim[9], Mark J. Siedner[2,4,5,10], Emily B Wong[2,11], Frank Tanser[2,12,13,14,15]

[1]*Digital Epidemiology Laboratory, Digital Futures, University of Cincinnati, Cincinnati, OH, USA*

[2]*Africa Health Research Institute, KwaZulu-Natal, South Africa*

[3]*Nelson R Mandela School of Medicine, University of KwaZulu-Natal, Durban, South Africa*

[4]*Division of Infectious Diseases, Massachusetts General Hospital, Boston, MA, USA*

[5]*Harvard Medical School, Boston, MA, USA*

[6]*International Institute for Rural Health, University of Lincoln, Lincolnshire, UK*

[7]*Department of Biostatistics and Bioinformatics, Rollins School of Public Health, Emory University, Atlanta, GA, USA*

[8]*Department of Biomedical Informatics, Emory University School of Medicine, Emory University, Atlanta, GA, USA*

[9]*Department of Population Health, New York University Grossman School of Medicine, New York, NY, USA*

[10]*School of Clinical Medicine, College of Health Sciences, University of KwaZulu-Natal, Durban, South Africa*

[11]*Division of Infectious Diseases, University of Alabama Birmingham, Birmingham, AL, USA*

[12]*Centre for Epidemic Response and Innovation (CERI), School of Data Science and Computational Thinking, Stellenbosch University, Stellenbosch, South Africa*

[13]*South African DSI-NRF Centre of Excellence in Epidemiological Modelling and Analysis (SACEMA), Stellenbosch University, Stellenbosch, South Africa*

[14]*School of Nursing and Public Health, College of Health Sciences, University of KwaZulu-Natal, Durban, South Africa*

[15]*Centre for the AIDS Programme of Research in South Africa (CAPRISA), University of KwaZulu-Natal, Durban, South Africa*

Supplementary Figures

Supplementary Figure 1. *Spatial location of the area of study illustrated in red in the Kwazulu-Natal province, South Africa*

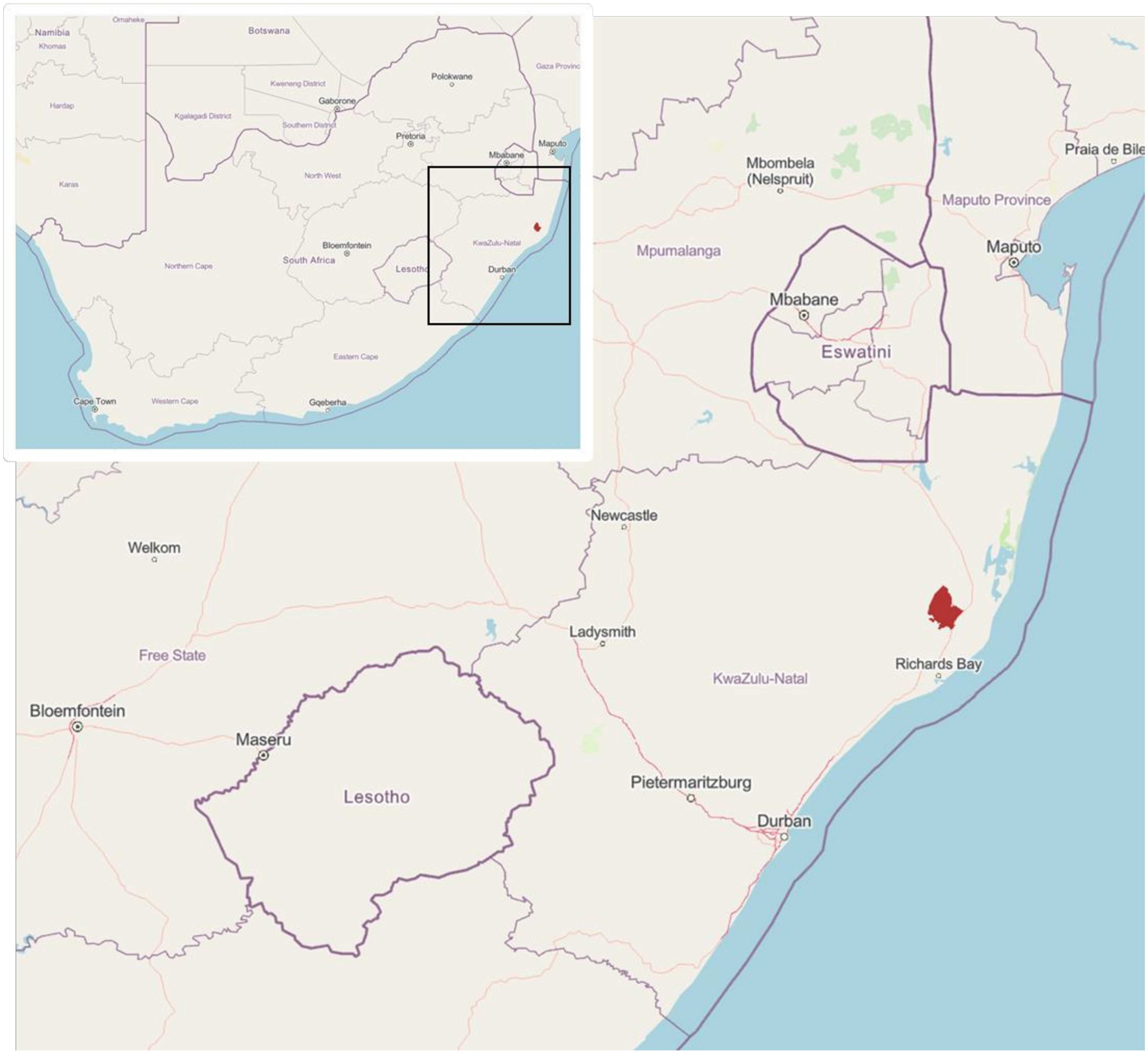

Supplement: Supplementary data [file bmjgh-2023-012730supp001.pdf]
