# Supplementary material for: Convergence of HIV and non-communicable disease epidemics: geospatial mapping of the unmet health needs in an HIV hyperendemic community in South Africa
Source: BMJ Glob Health. 2024 Jan 4;9(1):e012730. doi: 10.1136/bmjgh-2023-012730 (PMC10773360; doi:10.1136/bmjgh-2023-012730)
Supplement: Supplementary data [file bmjgh-2023-012730supp002.pdf]

Author Reflexivity Statement for: Convergence of HIV and non-communicable disease epidemics: Geospatial mapping of the unmet health needs in a HIV hyperendemic community in South Africa

Diego F Cuadros[1*], Chayanika Devi[1], Urisha Singh[2,3], Stephen Olivier[2], Alison Castle[2,4,5], Yumna Moosa[2], Johnathan A Edwards[6,7,8], Hae-Young Kim[9], Mark J. Siedner[2,4,5,10], Emily B Wong[2,11], Frank Tanser[2,12,13,14,15]

[1]*Digital Epidemiology Laboratory, Digital Futures, University of Cincinnati, Cincinnati, OH, USA*

[2]*Africa Health Research Institute, KwaZulu-Natal, South Africa*

[3]*Nelson R Mandela School of Medicine, University of KwaZulu-Natal, Durban, South Africa*

[4]*Division of Infectious Diseases, Massachusetts General Hospital, Boston, MA, USA*

[5]*Harvard Medical School, Boston, MA, USA*

[6]*International Institute for Rural Health, University of Lincoln, Lincolnshire, UK*

[7]*Department of Biostatistics and Bioinformatics, Rollins School of Public Health, Emory University, Atlanta, GA, USA*

[8]*Department of Biomedical Informatics, Emory University School of Medicine, Emory University, Atlanta, GA, USA*

[9]*Department of Population Health, New York University Grossman School of Medicine, New York, NY, USA*

[10]*School of Clinical Medicine, College of Health Sciences, University of KwaZulu-Natal, Durban, South Africa*

[11]*Division of Infectious Diseases, University of Alabama Birmingham, Birmingham, AL, USA*

[12]*Centre for Epidemic Response and Innovation (CERI), School of Data Science and Computational Thinking, Stellenbosch University, Stellenbosch, South Africa*

[13]*South African DSI-NRF Centre of Excellence in Epidemiological Modelling and Analysis (SACEMA), Stellenbosch University, Stellenbosch, South Africa*

[14]*School of Nursing and Public Health, College of Health Sciences, University of KwaZulu-Natal, Durban, South Africa*

[15]*Centre for the AIDS Programme of Research in South Africa (CAPRISA), University of KwaZulu-Natal, Durban, South Africa*

Author Reflexivity Statement

1. *How does this study address local research and policy priorities?*

Our study delves into the spatial overlap of unmet health needs for HIV, diabetes, and hypertension within a HIV hyperendemic community in KwaZulu-Natal, South Africa. By employing geospatial mapping techniques, we provide insights into the spatial distribution of these chronic health conditions and their unmet needs. Highlighting areas with the highest concentration of individuals with undiagnosed and uncontrolled conditions, our research emphasizes the need for targeted interventions in specific regions. In doing so, our study aligns with and contributes to local research and policy priorities, aiming to address the multifaceted health challenges faced by this South African community.

2. *How were local researchers involved in study design?*

Local researchers played a key role in the design of the Vukuzazi study, which is the source of the data analyzed in this study. Drawing from their in-depth knowledge of this KwaZulu-Natal community and its unique health challenges, they provided invaluable insights that shaped the objectives of the study and methodologies. Their familiarity with the socio-cultural dynamics of the region ensured that the research approach was culturally sensitive and contextually relevant. Furthermore, local researchers facilitated community engagement, ensuring that the design of the study was not only academically rigorous but also resonated with the lived experiences of the community members. Their involvement was instrumental in bridging the gap between academic research and on-ground realities, ensuring that the outcomes of the Vukuzazi study would be both actionable and impactful for the local community.

3. *How has funding been used to support the local research team?*

Funding for this study was used to support the local research team through capacity building, training, and resources necessary for data analysis. Financial support also facilitated travel and collaborations between local and international researchers, contributing to knowledge exchange and partnership strengthening.

4. *How are research staff who conducted data collection acknowledged?*

The publicly available data source from the Africa Health Research Institute (AHRI) was acknowledged in our manuscript.

5. *Do all members of the research partnership have access to study data?*

Data used in this study is publicly available from the Africa Health Research Institute (AHRI) database.

6. *How was data used to develop analytical skills within the partnership?*

Data played an important role in fostering analytical skills within our partnership. Local researchers underwent training in geospatial analysis techniques, enabling them to actively participate in data interpretation. This hands-on approach not only contributed to their skill development but also enriched the overall analytical process. Through regular meetings centered around data analysis and findings, we cultivated a collaborative learning environment. These sessions served as platforms for knowledge exchange, ensuring continuous capacity building and skill enhancement within the team.

7. *How have research partners collaborated in interpreting study data?*

Research partners from the US and South Africa actively collaborated in the data interpretation process. By pooling their expertise and diverse perspectives, they ensured a holistic understanding of the findings of the study. This collaborative approach was facilitated by consistent communication and regular meetings, which not only promoted a shared interpretation of the data but also enhanced the overall robustness and depth of the conclusions of the study.

8. *How were research partners supported to develop writing skills?*

To support the development of writing skills among research partners, early career and local researchers received mentorship from seasoned academics and researchers from the US. This collaboration offered them hands-on experience in manuscript drafting and revision. Through continuous feedback and guidance, the partnership ensured that these researchers not only honed their writing skills but also built their capacity to produce high-quality academic content.

9. *How will research products be shared to address local needs?*

To address local needs, the findings of the study are being made widely accessible by publishing in open-access journals and scientific conferences. This ensures that local researchers, policymakers, and other stakeholders in South Africa can easily access and utilize the information. Beyond publication, we have devised a dissemination plan that targets key stakeholders in South Africa, sharing crucial findings and recommendations with them. This proactive approach aims to directly influence and inform local policymaking and the development of relevant interventions.

10. *How is the leadership, contribution, and ownership of this work by LMIC researchers recognized within the authorship?*

The leadership, contribution, and ownership of LMIC researchers are prominently acknowledged through their positioning in the authorship list, which mirrors their roles in shaping the study from design to interpretation. The composition of the authorship, with a balanced representation of researchers from both South Africa and the US, stands testament to the equitable and collaborative nature of this international partnership.

11. *How have early career researchers across the partnership been included within the authorship team?*

Early career researchers from both South Africa and the US have been included in the authorship team, reflecting their contributions to the study and supporting their professional development.

12. *How has gender balance been addressed within the authorship?*

The authorship team includes a balanced representation of genders, recognizing the importance of diversity and inclusivity in research partnerships.

13. *How has the project contributed to the training of LMIC researchers?*

The project has been instrumental in enhancing the capabilities of LMIC researchers. They were actively involved in various stages, from study design to data interpretation, which fostered hands-on learning. Collaborative interactions with US researchers further enriched their experience, offering them training in advanced geospatial analysis techniques and refining their writing skills. This holistic approach ensured both capacity building and skill development among the LMIC research community.

14. *How has the project contributed to improvements in local infrastructure?*

While the project may not have directly led to tangible infrastructure enhancements, its findings offer invaluable insights for KwaZulu-Natal and potentially other similar regions. By mapping

the spatial overlap of unmet health needs for HIV, diabetes, and hypertension, the study highlights areas requiring targeted interventions and resources. These insights can guide local policymakers and stakeholders in prioritizing infrastructure investments, ensuring that healthcare facilities and services are optimally positioned to address the multifaceted health challenges faced by the community.

15. *What safeguarding procedures were used to protect local study participants and researchers?*

To protect the privacy and well-being of study participants, ethical approval was obtained from relevant institutional review boards, and informed consent was acquired from all participants included in the Vukuzazi study, the data source of our study.

Cuadros DF, *et al. BMJ Glob Health* 2024; 9:e012730. doi: 10.1136/bmjgh-2023-012730
